# Periodontal status and chronic obstructive pulmonary disease (COPD) exacerbations: a systematic review

**DOI:** 10.1186/s12903-021-01757-z

**Published:** 2021-09-03

**Authors:** Niamh Kelly, Lewis Winning, Christopher Irwin, Fionnuala T. Lundy, Dermot Linden, Lorcan McGarvey, Gerard J. Linden, Ikhlas A. El Karim

**Affiliations:** 1 Centre for Dentistry, School of Medicine Dentistry and Biomedical Sciences, Queen’s University Belfast, Belfast, UK; 2 Division of Restorative Dentistry and Periodontology, Dublin Dental University Hospital, Trinity College Dublin, University of Dublin, Lincoln Place, Dublin, Ireland; 3 The Wellcome-Wolfson Institute for Experimental Medicine, School of Medicine, Dentistry and Biomedical Sciences, Queen’s University Belfast, 97 Lisburn Road, Belfast, BT9 7BL UK; 4 Institute of Clinical Sciences Block B, Centre for Public Health, School of Medicine, Dentistry and Biomedical Sciences, Queen’s University Belfast, Belfast, UK

**Keywords:** COPD, Exacerbation, Periodontal disease, Oral bacteria, Oral health

## Abstract

**Background:**

A growing body of evidence suggests a role for oral bacteria in lung infections. This systematic review aimed to analyse the association between poor periodontal status and the frequency of chronic obstructive pulmonary disease (COPD) exacerbations.

**Methods:**

PubMed, Embase, Web of Science, CINAHL and Medline were searched for studies published until May 2020, with no language restriction. Studies reporting periodontal condition, or periodontal treatment outcomes, with data on the frequency of exacerbations of COPD, were identified. The primary outcome was the frequency of exacerbations and secondary outcomes included quality of life (QoL) and hospitalisation. Quality and risk of bias assessment were carried out using the Newcastle Ottawa Scale for observational studies, Robins-1 tool for non-randomised intervention studies and Cochrane risk of bias assessment (RoB-2) tool for randomised clinical trials. Studies were assessed for eligibility and quality by two assessors independently.

**Results:**

Searches identified 532 records and 8 met the inclusion criteria. Included studies were three clinical trials, one prospective cohort study, one case–control, and three cross-sectional studies. A narrative synthesis was performed. The data from intervention studies showed reduction in the frequency of exacerbations following periodontal treatment. Data from observational studies suggest association of worse plaque scores and fewer teeth with exacerbation, but not pocket depth or clinical attachment loss. Better periodontal health was also associated with reduced frequency of COPD exacerbations, hospitalisations and improved quality of life in COPD patients. Due to the high heterogeneity no meta-analysis was performed. The quality of some of the included studies was low and there was evidence of a high risk of bias.

**Conclusion:**

The data supports possible association between poor periodontal health, the frequency of exacerbations, hospitalisation and quality of life in COPD patients. The evidence is of moderate to low certainty and is limited by high risk of bias suggesting the need for well-designed and adequately powered randomised controlled trials, to inform future research and clinical practice.

*The PROSPERO registration number* CRD42020180328.

**Supplementary Information:**

The online version contains supplementary material available at 10.1186/s12903-021-01757-z.

## Background

Periodontitis is defined as a chronic multifactorial disease associated with dysbiotic plaque biofilms, characterised by loss of periodontal support, clinical attachment loss, gingival bleeding, periodontal pocketing and alveolar bone loss [1]. Emerging evidence suggests oral bacteria and local inflammatory response in periodontal tissues contribute to systemic inflammation and increase the risk for development of chronic inflammatory conditions including diabetes, cardiovascular and respiratory disease [2–5].

Chronic obstructive pulmonary disease (COPD) is a common preventable and treatable respiratory disease characterised by persistent airflow limitation that is usually progressive and associated with an enhanced chronic inflammatory response in the airways and the lungs to noxious particles or gases [6]. It has a worldwide prevalence of 9–10% in adults > 40 years of age and is responsible for an estimated global annual death toll of 3 million [7]. It is well recognised that smoking is the primary risk factor for COPD [7], but emerging evidence suggests that periodontitis is associated with increased risk of development of COPD [8, 9]. COPD and periodontitis share several risk factors such as age, smoking, stress and ethnicity [10]. The diseases also have similar pathophysiology, characterised by inflammation, recruitment of neutrophils and release of proteolytic enzymes, resulting in the destruction of the pulmonary alveolus or destruction of the periodontal tissues [11]. Patients with confirmed COPD have lower tooth brushing frequency and poorer periodontal health than comparable control groups [12, 13]. The association between periodontitis and COPD has been the subject of several observational studies [14, 15], including a longitudinal study [9]. In a meta-analysis of 14 observational studies, periodontal disease was found to be a significant and independent risk factor for COPD, however, whether a causal relationship exists remains uncertain [16].

Progressive lung function decline may be accelerated by acute exacerbations of COPD (AE-COPD) [7]. These acute episodes frequently necessitate additional therapy and may also lead to hospitalisation incurring substantial healthcare costs. Factors that contribute to AE-COPD include co-morbidities, smoking, airway infections (bacterial and viral) and environmental pollution. Studies have shown that bacterial lung infections are the cause of 50% of COPD exacerbations [17]. The majority of AE-COPD respond to antibiotic treatment, providing further evidence that infection is an important factor [18]. Increased microbial diversity in COPD patients has been demonstrated with the identification of oral bacteria in their lung microbiome and tissue [19, 20]. In COPD patients, it is possible that reduced laryngotracheal mechanosensitivity and decreased airway clearance due to impaired mucociliary function [21, 22], increases the risk of aspiration of oral secretions and bacteria.

One of the suggested mechanisms through which poor oral health and periodontal disease contribute to the development and progression of COPD is by aspiration of pathogenic bacteria [23]. The dental plaque biofilm, particularly that associated with the tissue changes in periodontal disease, incorporates pathogenic bacterial species that may be disseminated to cause infection in extra-oral sites [24, 25]. Poor oral hygiene may contribute to the colonisation of dental plaque by respiratory pathogens and elevated antibody levels against key periodontal pathogens including *Fusobacterium nucleatum* and *Prevotella intermedia* have also been found in the sputum of patients with an acute exacerbation of chronic bronchitis, further supporting a role for oral bacteria in lung infections [26].

Frequent AE-COPD is associated with accelerated lung function decline, decreased quality of life, increased mortality rates and poorer survival outcomes, thereby placing a significant burden on health care services [11, 27]. Therefore, strategies to prevent or reduce the frequency of COPD exacerbations are required. We hypothesise that improvement in periodontal health could reduce the frequency of AE-COPD. While there are suggestions of an association [28], there is currently no clear evidence on the strength of any association between periodontal disease and COPD exacerbations to inform clinical practice. A number of systematic reviews reported on the association between periodontal and respiratory disease including COPD [5, 16, 29], however, to our knowledge there has not been a systemic review with a research question that focused on the association of the periodontal disease with COPD exacerbations. This systematic review aims to critically appraise the emerging literature and to synthesise evidence on a putative link between poor periodontal health and COPD exacerbations to inform research and clinical practice. The objectives of the review are to address the following research questions: “Is poor periodontal health associated with a higher frequency of AE-COPD”? and “Does periodontal treatment lead to a reduction in AE-COPD events in patients with COPD.

## Methods

### Protocol registration and focused question

This systematic review is reported using PRISMA guidelines and the PECO/PICO framework and registered in PROSPERO registration number CRD42020180328. The aim of the review is to address two questions:

1. In adult patients with COPD (P) is poor periodontal status (E) associated with a higher frequency of AE-COPD (O) compared to good periodontal status (C)?
2. In adult patients with COPD (P) does periodontal treatment (I) reduce AE-COPD events (O) compared to no treatment (C)?

### Eligibility criteria

The studies eligible for inclusion were randomised clinical trials, cross-sectional studies, retrospective case control studies and cohort studies. Studies were considered if they included adult participants (≥ 18yrs) diagnosed with COPD, provided details of acute exacerbations of COPD and /or QoL, and included an assessment of the periodontal status including periodontal disease indices, poor oral hygiene and tooth loss. Animal studies, non-clinical research, expert opinion, reviews, and studies not available in full text version were excluded.

**Fig. 1:**
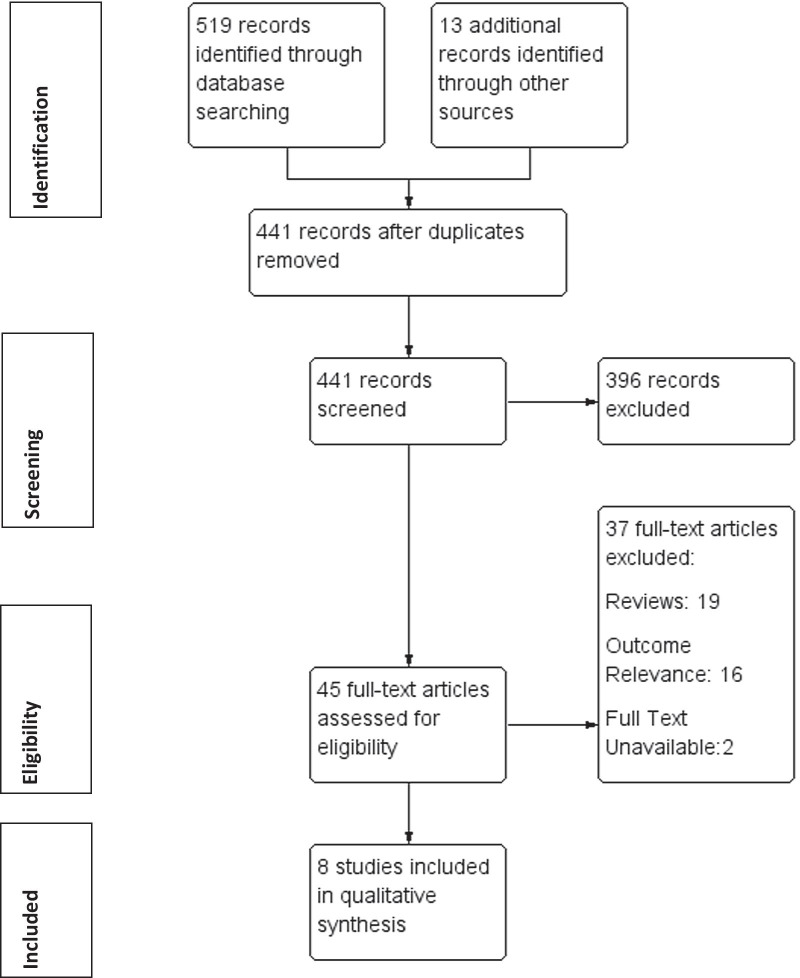
PRISMA flow diagram

The primary outcomes were increased frequency of AE-COPD associated with poor periodontal health or reduced frequency AE-COPD as a result of improved periodontal health in response to treatment. Secondary outcomes included quality of life, increase/reduction in hospital admissions and treatment costs. The PRISMA flow chart (Fig. 1) illustrates the selection process. For screening and assessment of eligibility criteria, titles and abstracts were screened by two assessors independently (NK, IEK). Full texts were obtained for all studies that met the inclusion criteria or when the abstract did not contain sufficient information to decide on the selection criteria. Full-text articles were assessed independently for inclusion in the review by three assessors (NK, LW, and IEK).

### Information sources and search strategy

Electronic database searches were undertaken using a combination of key search words (chronic obstructive pulmonary disease, exacerbation, reduced lung function, hospitalisation(s), quality of life, oral hygiene, periodontitis, and gingivitis). These MESH search items and search strategy (Additional file 1: Table 1) were developed for the MEDLINE search and adopted for other electronic databases. Medline, Embase, Web of Science and CINAHL were searched from inception to May 2020 with no language restriction. We developed and optimised the search strategy for Pubmed and the same was used for other databases. To ensure literature saturation, reference lists of included studies were checked for eligible studies and the grey literature was searched using OpenGrey.

### Quality assessment of included studies

For randomized trials, the Cochrane modified risk of bias tool (RoB 2.0) was used to assess the quality of studies. The studies were categorized as having ‘low’, ‘some concerns’ or ‘high risk’ of bias based on the following domains: randomization, deviations from intended interventions, missing outcome data, measurement of the outcome, selection of the reported result and overall quality [30]. The methodological quality of non-randomised studies was assessed using Robins-1 tool and the Newcastle–Ottawa scale for case–control studies and cohort studies an adaptation of this scale [31] for cross-sectional studies. The risk of bias and quality of studies was assessed independently by three assessors (NK, LW, IEK). Furthermore, the certainty of evidence in each of the outcomes was assessed using the Grading of Recommendations Assessment, Development and Evaluation (GRADE) evidence quality assessment tool [32]. We assessed the certainty of the body of evidence with reference to the overall risk of bias of the included studies, the directness of the evidence, the consistency of the results, the precision of the estimates, and the risk of publication bias. We classified the certainty of the body of evidence into four categories: high, moderate, low and very low.

### Data extraction and analysis

Data were extracted using custom-designed forms (adopted from the Cochrane library). Extracted data included; the type of study, number and demographics of participants, COPD diagnosis, periodontal health parameters, respiratory outcomes, intervention/exposure, funding source, duration of follow-up, location of the study, quality of life assessment and hospitalisation. The final data included for analysis were agreed by three authors (NK, LW, IEK) and any differences of opinion were resolved by further discussion. Due to the heterogeneity of study designs, no meta-analysis or calculation of I^2^ was performed. A narrative synthesis of included studies outlining the primary outcome (frequency of COPD exacerbations) and secondary outcomes (QOL and hospitalisations) was included.

## Results

### Study selection

The search strategy identified 532 original titles and abstracts that were screened for potential eligibility, from which 45 full-texts were screened for inclusion (Fig. 1). Eight articles met the inclusion criteria for the review, three cross-sectional studies Zhou et al*.* [33], Liu et al*.* [34], AbdelHalim et al*.* [35], one case–control study Baldomero et al. [36], one prospective cohort study Barros et al*.* [37] and three clinical trials Zhou et al. [38], Kucukcoskun et al. [39] and Agado et al*.* [40]. A full description of the included studies and their characteristics are outlined in Table 1. The excluded studies and the reason for exclusion are cited and outlined in Additional file 1: Table 2.

**Table 1:** Studies and population characteristics

References	Type of study	Location	Number/gender	Age (years)	Follow-up	Periodontal assessment	Intervention exposure	COPD diagnosis	Funding
Zhou et al. [33]	Cross-sectional	China	306 210 M 96 F	Mild: 65.5 ± 8.62 Mod: 64.4 ± 10.2 Severe: 63.1 ± 9.24 Very Severe:62.3 ± 11.0	None	Periodontal examination (PI, BI, PPD, CAL) and Number of teeth Diagnostic criteria not reported	Poor periodontal health	GOLD Criteria	ISTCRG NNSFC
Liu et al. [34]	Cross-sectional	China	392 287 M 105 F	Cases: 64.3 ± 10.1 Controls: 63.6 ± 9.7	None	Periodontal examination (PI, BI, PPD, CAL) Diagnostic criteria was: PD > 3 mm, CAL > 4 mm used to diagnose periodontal disease Interview re oral hygiene behaviours	Poor periodontal health	GOLD Criteria	NNSFC NSFB BSTPF
Agado et al. [40]	Randomised control trial	USA	30 20 M 10 F	UI:62 ± 7.76 HI:68 ± 7.20 Control:62 ± 8.33	None	Periodontal examination (PI, CAL) Diagnostic criteria was: > 6 teeth and minimum mean CAL of 1.5 mm used to diagnose periodontal disease	Periodontal treatment	Not stated	ISUIM
Barros et al. [37]	Prospective cohort	USA	1635 930 M 705F	Events (No): 66.0 (5.13); Events (Yes): 63.9 (5.69)	5 years	Periodontal examination (PPD, CAL)**	Periodontal health/edentualism	GOLD Criteria	GSK
Kucukcokun et al. [39]	Clinical trial	Turkey	40 35 M 5 F	Intervention: 61.8 ± 7.57 Control: 57.85 ± 12.09	1 year	Periodontal examination (PI, GI, PPD, BOP and CAL)^#^	Periodontal treatment	GOLD Criteria	NS
Zhou et al. [38]	Randomised control trial	China	60 47 M 13 F	Intervention: 63.9 ± 9.44, 65.3 ± 7.54 Control: 68.0 ± 7.64	2 years	Periodontal examination (PI, BI, PPD, CAL)*	Periodontal treatment	GOLD Criteria	NNSFC
AbdelHalim et al. [35]	Cross-sectional	Egypt	250 250 M 0 F	Cases: 56.75 ± 10.42 Controls: 55.28 ± 9.12	None	Periodontal examination (PI, BI GI, PPD and CAL)^##^	Poor periodontal health	GOLD Criteria	NS
Baldomero et al. [36]	Case–control	USA	136 136 M 0 F 56 had dental exam	Cases: 66.8 (7.2) Controls: 67.5 (5.5)	None	Oral health questionnaire & Periodontal examination (PI, BOP, GI, PPD and CAL)^##^	Poor periodontal health	ACP, ACCP and ATS	NHLBI VACDA

PI, plaque index; BI, bleeding index; BOP, bleeding on probing; GI, gingival index; PPD, probing pocket depth; CAL, clinical attachment level); N/A, Not available; QoL, quality of life; SGRQ, St George’s Respiratory Questionnaire; OHIP, 5-Oral health impact profile-5; GOLD, Global Initiative for Obstructive Lung Disease; ACP, American College of Physicians; ACCP, American College of Chest Physicians; ATS, American Thoracic Society criteria; NHLBI, National Heart, Lung and Blood Institute; VACDA, Veterans Affairs Career Development Award; GSK, GlaxoSmithKline; NNSFC, National Natural Science Foundation of China; NSFB, Natural Science Foundation of Beijing; BSTPF, Beijing Science and Technology Programme Fund; NS, not stated; ISTCRG, International Science and Technology Cooperation Research Grant, Beijing Municipal Science and Technology Commission; ISUIM, Idaho State University Intra Mural

**JCDC + AAP-joint Center for Disease Control/American Association of Periodontology

^#^1999 AAPW-1999 American Academy of Periodontology Workshop guidelines

^##^AAPTFR- American Academy of Periodontology Task Force Report

### Assessment of risk of bias in the included studies

Risk of bias assessment of clinical trials showed an overall high risk of bias in Zhou et al. [38] and Agado et al*.* [40], but moderate in Kucukcoskun et al. [39] as assessed by Robins-1 tool (Additional file 1: Fig. 1). Three of the observational studies, Zhou et al*.* [33], Liu et al*.* [34] and AbdelHalim et al*.* [35] were assessed to be of fair quality and that of Baldemero et al*.* [36] and Barros et al*.* [37] were assessed to be of good quality (Additional file 1: Table 3A-C).

### Periodontal health and frequency of COPD exacerbations

Three observational studies (two cross-sectional studies and one case–control study) assessed the frequency of COPD exacerbations related to periodontal disease and measures of periodontal health. Different case definitions for periodontal disease have been used in different studies as shown in Table 1. AbdelHalim et al. [35] found that frequent exacerbation is associated with high plaque index (PI) scores, high Bleeding Index (BI), high Gingival Index (GI) and moderate to severe clinical attachment loss (CAL) and probing pocket depth (PPD). In a regression model to predict the influence of periodontal health variables on the frequency of exacerbation, GI was found to be the most important predictor of exacerbations followed by high PI. However, when adjusting for demographics, clinical parameters, C-Reactive Protein level and spirometry data, only PI and BI remained significantly associated with exacerbations. It is not clear however if the model adjusted for common confounders such as smoking.

Liu et al. [34] on the other hand found no significant difference in PPD, CAL and BI between the frequent and infrequent exacerbation groups. But fewer remaining teeth, high PI scores, low tooth brushing times, and low regular supra-gingival scaling were significantly associated with COPD exacerbations. After adjustment for age, gender, smoking and body mass index, fewer remaining teeth, high PI scores and low tooth brushing time were significantly associated with exacerbations. With additional adjustment for COPD and dyspnoea severity only fewer remaining teeth and low tooth brushing times remained statistically significant. In a case–control study, Baldomero et al. [36] used logistic regression models to estimate the associations between oral health and COPD exacerbation status, taking into account potential confounders including inhaler use and forced expiratory volume in the first second (FEV1) % predicted. They found that unadjusted and adjusted odds ratio for self-reported oral health status and dental exam measures did not vary significantly between exacerbators and non-exacerbators. Results of individual studies statistical analysis findings are outlined in Table 2.

**Table 2:** Results summary for the primary outcome COPD exacerbation frequency

Study	Outcome and how measured	Results summary
Liu et al. [34]	Self-reported COPD exacerbations/change in clinical symptoms and medication	CAL, PPD and BI were not associated with exacerbations. However, fewer remaining teeth [OR = 1.69, 95%CI 1.03–2.77, *p* = 0.04], and low tooth brushing times [OR = 4.19, 95% CI 1.44–12.1, *p* = 0.008] were significantly associated with exacerbations after adjusting for age, gender, smoking, body mass index, COPD severity and dyspnoea scores
Kucukcoskun et al. [39]	COPD exacerbations, confirmed by chest physician	Periodontal treatment result in significant reduction in GI (*p* = 0.002), PPD (*p* = 0.003), CAL (*p* = 0.001), and BOP (*p* = 0.002) at month 6. At 12 month follow up, exacerbation frequency was significantly reduced in those who received periodontal treatment (*p* = 0.01). Sex, age, FEV1, FVC, COPD severity, number of previous exacerbations, and PPD > 4 mm did not affect exacerbation frequency
Zhou et al. [38]	Self-reported COPD exacerbations/ change in clinical symptoms and medication/spirometry	At the 2-year follow up, periodontal treatment groups had a lower proportion of frequent exacerbations (SRP 30%, supra-gingival scaling 15.8%) compared with the control group (66.7%) and the difference was statistically significant (*p* = 0.004). After adjusting for age, gender body mass index, smoking status, and baseline frequent exacerbations the ORs for frequent COPD exacerbation were 0.29 (95% CI 0.10–0.84) for the SRP group and 0.04 (95% CI 0.003–0.64) for the scaling group
AbdelHalim et al. [35]	Self-reported COPD exacerbations and spirometry measurements	Frequent exacerbations were associated with high PI scores (*p* = 0.029), high BI (*p* = 0.04), high GI (*p* < 0.001), moderate to severe clinical attachment loss (CAL) and probing pocket depth (PPD) (*p* < 0.001). GI appeared to be the most important predictor of exacerbations (*P* < 0.001) followed by PI (*P* = 0.05). Adjusting for demographics, clinical parameters, C–reactive protein level and spirometry data only PI (*p* = 0.003) and BI (*p* = 0.04) remained significantly associated with exacerbations
Baldomero et al. [36]	Self-reported COPD exacerbations verified by medical chart review	The unadjusted and adjusted odds ratio for self-reported oral health status and dental exam measures did not vary significantly between exacerbators and non-exacerbators. There was a trend towards higher odds of exacerbations in those with “dry mouth” in both unadjusted [OR 2.18; 95% CI 1.09–4.43, *p* = 0.03] and adjusted [OR 2.29; 95% CI 0.99–5.44, *p* = 0.05] models

### Periodontal treatment and frequency of COPD exacerbations

Two intervention studies that investigated the effect of periodontal treatment on the frequency of exacerbations in patients with COPD were identified. Zhou et al*. *[38] and Kucukcoskun et al*.* [39] found that non-surgical periodontal treatment improved lung function and decreased the frequency of exacerbations in COPD patients.

Zhou et al*.* [38] tested the effect of supra-gingival scaling alone, scaling and root planing (SRP) and no treatment on the frequency of exacerbations. They found that periodontal treatment resulted in improvement in periodontal measures including, PPD, CAL, BI, and PLI in comparison with no treatment. There were no statistically significant differences among the three groups for the frequency of exacerbation at baseline, but frequent exacerbation decreased in both the supragingival scaling group and SRP group, compared to the control group. At the 2-year follow up, the treatment groups had a lower proportion of frequent exacerbations compared to the control group. The difference between the intervention and control groups was significant after adjusting for age, gender body mass index, smoking status, and baseline frequent exacerbations.

In a non-randomised trial Kucukcoskun et al. [39], compared the effect of periodontal treatment compared to no treatment on the frequency of exacerbations at 12 months follow up. There were no significant differences in the periodontal parameters of the control and the intervention group at baseline, but periodontal treatment resulted in a significant reduction in periodontal parameters at month 6. After 12 months follow up they found that exacerbation frequency was significantly reduced in the treatment group. They also found that the median exacerbations declined from 3 to 2 in the test group, and increased from 2 to 3 in the control group.

### Periodontal health and quality of life in COPD patients

Three studies assessed periodontal health and the quality of life in COPD patients. Zhou et al*.* [33] and Baldomero et al*.* [36]) showed an association between better periodontal health and the quality of life of COPD patients. Baldomero et al*.* [36] found that worse Oral Health Impact Profile-5 (OHIP-5) scores were strongly associated with worse St George’s respiratory questionnaire (SGRQ) scores, used to assess the quality of life related to respiratory health status. Zhou et al*.* [33] found that among the periodontal parameters tested and after adjusting for confounding factors, only missing teeth and high PI scores were significantly associated with poorer quality of life in COPD patients assessed by SGRQ. Agado et al*.* [40] on the other hand found that non-surgical periodontal treatment for chronic periodontitis did not affect the quality of life and illness in patients with COPD. Details of the results of these studies are summarised in Table 3.

**Table 3:** Summary of secondary outcomes of hospitalisations and quality of life

Study	Outcome and how measured	Results summary
Zhou et al. [33]	QoL assessed by SGRQ scores	After adjusting for age, gender, body mass index, and smoking status, missing teeth was significantly associated with symptom score (*p* = 0.030) and activity score (*p* = 0.033) and plaque index was significantly associated with symptom score (*p* = 0.007)
Agado et al. [40]	QoL measured by SGRQ-A and self-assessment of overall current health in COPD patients receiving periodontal treatment and control group with no treatment	SGRQ–A and Illness Questionnaire scores showed no significant differences between groups in quality of life or illness following periodontal treatment. Total SGRQ scores decreased among groups but not significantly (*p* = 0.138)
Barros et al. [37]	Frequency of COPD-related events and hospitalisation from hospital records	Periodontal disease and edentulism are associated with increased risk of COPD event requiring hospitalisation but after adjusting for age, race, centre, gender, education, hypertension, BMI and smoking only edentulism remained significant (Hazard Ratio 2.28, 95% CI 1.46–3.56)
Kucukcoskun et al. [39]	Hospitalisation confirmed by a physician	There were 7 hospitalisations in the test group and 12 in the control group over 12 months of follow-up
AbdelHalim et al. [35]	Self-reported number of hospitalisations	All periodontal health parameters correlate significantly with the number of hospitalisations per year (*p* < 0.001)
Baldomero et al. [36]	St. George’s Respiratory Questionnaire (SGRQ) and OHIP-5), hospitalisations	There was a strong association between OHIP-5 items, difficulty chewing (OR 2.57, *p* = 0.02), painful ache in the mouth (OR 5.4, *p* < 0.001), appearance (OR 3.1, *p* = 0.003), less flavour (OR 3.5, *p* = 0.005) and difficulty doing jobs (OR 7.3, *p* < 0.001) worse respiratory health scores after adjusting for inhaler use and FEV1% predicted There was a non-significant trend towards more severe COPD exacerbations requiring emergency room visits and/or hospitalisations in those with worse periodontal health indices

### Periodontal health and risk of hospitalisation in COPD patients

Four studies (three observational and one clinical trial) included an assessment of hospitalisation frequency as a result of COPD exacerbations (Table 3). AbdelHalim et al*.* [35] reported that periodontal health parameters were significantly associated with the number of hospitalisations. In a large prospective cohort study, Barros et al. [37] found that periodontal disease and edentulism are associated with increased risk of hospitalisation but after adjusting for multiple covariates, only edentulism remained significant. Baldomero et al*.* [36] concluded that while those affected by COPD with poorer periodontal examination outcomes had an increased risk of hospitalisation or emergency department visits, compared to those with better periodontal status, this did not reach statistical significance. Kucukcoskun et al*.* [39] reported that the number of hospitalizations was seven in the test group and 12 in the control group during the 12 month follow-up period, but it is not clear if there is a statistically significant difference between the groups.

### Periodontal status and treatment costs for COPD patients

One of the secondary outcomes of this systematic review was to assess the link between periodontal status and treatment costs for COPD patients. However, the literature searches did not reveal any studies investigating this association.

### Assessment of the evidence certainty using GRADE

The GRADE evidence quality assessment tool was used to assess the certainty of the evidence for each of the outcomes. There was moderate certainty evidence from two clinical trials that periodontal treatment reduces the frequency of exacerbations. A moderate certainty evidence from three observational studies suggest poor periodontal health increases the frequency of exacerbations. Low certainty evidence from one clinical trial suggests that periodontal treatment has no effect on QoL but similar evidence from two observational studies suggest an association between poor periodontal health and QoL in COPD patients. There was low certainty evidence from three observational studies and low certainty evidence from one clinical trial suggest that poor periodontal health is associated with an increased risk of hospitalisation (Table 4).

**Table 4:** Summary findings and evidence certainty for all outcomes using GRADE

Outcomes	Impact	№ of participants (studies)	Certainty of the evidence(GRADE)
Exacerbations	Periodontal treatment results in a significant reduction in the frequency of exacerbations at one and 2 years follow	100 (one randomised and one non randomised trial)	⨁⨁⨁◯ (a) MODERATE
Exacerbations	Periodontal health parameters mainly PI, GI, BI, as well as fewer number of teeth and lower brushing times, are significantly associated with exacerbations	698 (3 observational studies)	⨁⨁⨁◯ (b) MODERATE
Quality of Life	Periodontal treatment did not improve the quality of life of COPD patients	30 (1 clinical trial)	⨁⨁◯◯ (a, c) LOW
Quality of Life	Worse OHIP 5, missing teeth and high PI scores were associated with worse SGRQ Scores	362 (2 observational studies)	⨁⨁◯◯ (b) LOW
Hospitalisation	There are 7 hospitalisations in the test compared to 12 in the control group at 12 months follow up	40 (1 Clinical trial)	⨁⨁◯◯ (a, c) LOW
Hospitalisation	Edentulous, poor oral hygiene, periodontal parameters PI, GI, BI are associated with increased risk of hospitalisation	1941 (3 observational studies)	⨁⨁◯◯ (d) LOW

GRADE Working Group grades of evidence

High certainty: We are very confident that the true effect lies close to that of the estimate of the effect

Moderate certainty: We are moderately confident in the effect estimate: The true effect is likely to be close to the estimate of the effect, but there is a possibility that it is substantially different

Low certainty: Our confidence in the effect estimate is limited: The true effect may be substantially different from the estimate of the effect

Very low certainty: We have very little confidence in the effect estimate: The true effect is likely to be substantially different from the estimate of effect

(a) Downgraded due to high risk of bias on randomisation and selection of reported results

(b) Observational studies upgraded due to effect size

(c) Downgraded due lack of precision (small sample size)

(d) Observational studies graded as low

## Discussion

Acute exacerbations are the key risk factor for the progression of COPD [41] and severe exacerbations that result in hospital admission are associated with high mortality levels [42, 43]. Therefore, identifying modifiable risk factors is important to help reduce the frequency of exacerbations and improve COPD treatment outcomes. The findings of this systematic review showed poor periodontal health and poor oral hygiene are associated with COPD exacerbations. The review also found that periodontal treatment was associated with a reduction in the frequency of COPD exacerbations. The findings are in agreement with previous studies which highlighted a potential relationship between periodontitis and respiratory function [3, 4, 16]. COPD and periodontitis are believed to have similar pathophysiology, as both diseases are characterised by chronic inflammation and shared risk factors [44]. Given the previously demonstrated role for oral bacteria in lung infections and pneumonia [25, 29], it is reasonable to suggest that improved oral health will have a positive impact on COPD patients.

Many studies have suggested a putative link between oral health and COPD exacerbations, but to answer our focused research questions we limited studies to those with a clinical diagnosis of COPD and clear, measurable indicators of periodontal health. The studies identified were, however, heterogeneous in terms of designs and measures of outcomes, in particular for secondary outcomes such as quality of life and hospitalisation, which necessitated a narrative synthesis for these outcomes.

For the primary outcome, it appears that higher plaque scores were associated with an increased frequency of COPD exacerbations for the majority of included studies, except for Baldomera et al. [36]. Moreover, the included clinical trials, Zhou et al*.* [38] and Kucukcoskun et al*.* [39] concluded that periodontal therapy in COPD patients improved lung function and resulted in decrease in the frequency of COPD exacerbations. Improving oral health by treating periodontal disease, which in turn improved plaque scores, also showed a similar trend. Data from the only two intervention studies available Kucukcoskun et al. [39] and Zhou et al. [38] supported an association between improvements in periodontal health resulting from treatment and a reduction in exacerbations during at least one year of follow-up.

These results are however not unexpected, as evidence for a link between oral bacteria and pneumonia is strong [5, 29]. The dental plaque biofilm may be a source of microorganisms associated with lung infections [24] and it is possible that in COPD patients with poor oral hygiene and high plaque scores, bacteria will be aspirated into the lungs leading to exacerbations [14]. In addition to teeth, respiratory pathogens has also been shown to colonise dentures [12] and denture wear to be associated with increased risk of pneumonia [45]. The findings that loss of teeth and edentulism reported in this review to be associated with AE-COPD could be attributable to denture use as a significant number of those with no or reduced number of teeth wear dentures [37].

Other important outcomes investigated in this review were quality of life and the frequency of hospitalisation related to exacerbations. The format in which the data for these outcomes was reported prevented meta-analysis, but generally, most studies suggested that poor periodontal status was associated with reduced quality of life and increased hospitalisation rate for COPD patients. The evidence also suggests providing periodontal treatment and improving periodontal health reduced the frequency of hospitalisations and improved the quality of life for COPD patients in studies analysing these outcomes.

To our knowledge, this is the first systematic review to analyse the link between periodontal status and the frequency of COPD exacerbations. The review was set to answer a specific question and followed standard systematic review methodology with clear inclusion and exclusion criteria. One of the limitations of the review, however, is the small number of included studies and aspects of the quality, particularly for the intervention studies. Additional limitations included the variability in COPD diagnostic criteria and the methods used to assess periodontal disease. Also, due to the small number of studies included, it was not possible to detect publication bias. Adjustment for covariates is essential to reducing the confounding effects in non randomised studies and  many of the included studies adjusted for various co-variates such demographics, smoking, BMI and use of inhaled corticosteroids and anticholinergic drugs. This however was not consistent across the studies and therefore these results should be interpreted with caution. Nevertheless, the review enhances the current body of knowledge and provides evidence that further research is required in this area.

AE-COPD is one of the leading causes of mortality worldwide [40] and places a significant financial burden on the health sector [41]. As oral health is a potentially modifiable risk factor, in theory improving oral health will reduce disease burden, but high certainty evidence on the association of AE-COPD with oral health is required to confirm risk factors and  change to clinical practice. Further studies should address the limitations of the current evidence particularly in relation to the design, power and conduction of randomised control trials and longitudinal studies.

## Conclusion

In conclusion, the findings of this systematic review suggest a potential link between poor periodontal status and reduced number of teeth and the frequency of COPD exacerbations. Qualitative evidence also highlights a potential positive correlation between improved periodontal health and a reduction in hospitalisation and improved quality of life in COPD patients. However, questions remain due to the high risk of bias, the low quality of some of the included studies and low to moderate certainty of the evidence. Well designed, adequately powered randomised controlled trials are needed to establish whether the periodontal condition influences the frequency of COPD exacerbations.

## Supplementary Information

**Additional file 1**. Supplementary data to include: **Supp Table 1**: detailed search strategy; **Supp Table 2**: details of excluded studies; **Supp Table 3**: Quality assessment of observational studies using Newcastle Ottawa Scale (NOS): 3A - adapted NOS for cross-sectional studies, 3B- NOS for case-control studies and 3C NOS for cohort studies. The domains covered by the scale included selection, comparability, outcomes, and exposure. Each asterisk represents whether the individual criterion within the subsection was fulfilled. A maximum of 9 can be assigned for each study using the Newcastle Ottawa Scale. A maximum score of 10 can be assigned for the adapted Newcastle Ottawa Scale for cross sectional studies. **Supp Figure 1**: Quality assessment of intervention studies using Cochrane RoB 2- tool and **Supp Figure 2**: Risk of bias assessment of non-randomised intervention studies using ROBINS-I tool.

## Data Availability

The datasets used and/or analysed during the current study are available from the corresponding author on reasonable request.

## Abbreviations

AE-COPD: Acute exacerbations in chronic obstructive pulmonary disease
COPD: Chronic obstructive pulmonary disease
CAL: Clinical attachment loss
FEV1: Forced expiratory volume in the first second
PPD: Probing pocket depth
PI: Plaque index
SGRQ: St George’s respiratory questionnaire
OHIP-5: Oral health impact profile-5
RoB: Risk of bias
