# Supplementary material for: Periodontal status and chronic obstructive pulmonary disease (COPD) exacerbations: a systematic review
Source: BMC Oral Health. 2021 Sep 3;21:425. doi: 10.1186/s12903-021-01757-z (PMC8418022; doi:10.1186/s12903-021-01757-z)
Supplement: Supplementary file 1 — Additional file 1. Supplementary data to include: Supp Table 1: detailed search strategy; Supp Table 2: details of excluded studies; Supp Table 3: Quality assessment of observational studies using Newcastle Ottawa Scale (NOS): 3A - adapted NOS for cross-sectional studies, 3B- NOS for case-control studies and 3C NOS for cohort studies. The domains covered by the scale included selection, comparability, outcomes, and exposure. Each asterisk represents whether the individual criterion within the subsection was fulfilled. A maximum of 9 can be assigned for each study using the Newcastle Ottawa Scale. A maximum score of 10 can be assigned for the adapted Newcastle Ottawa Scale for cross sectional studies. Supp Figure 1: Quality assessment of intervention studies using Cochrane RoB 2- tool and Supp Figure 2: Risk of bias assessment of non-randomised intervention studies using ROBINS-I tool. [file 12903_2021_1757_MOESM1_ESM.docx]

**Title:** Periodontal status and chronic obstructive pulmonary disease (COPD) exacerbations: A systematic review

**Author names and affiliations:**

Niamh Kelly ^1^, Lewis Winning ^2^, Christopher Irwin ^1^, Fionnuala T Lundy ^3^, Dermot Linden ^3^, Lorcan McGarvey ^3^, Gerard J Linden ^4^ and Ikhlas A El Karim ^3^

^1^ Centre for Dentistry, School of Medicine Dentistry and Biomedical Sciences, Queen’s University Belfast, Belfast, UK.

^2^ Division of Restorative Dentistry & Periodontology, Dublin Dental University Hospital, Trinity College Dublin, University of Dublin, Lincoln Place, Dublin, Ireland.

^3^ The Wellcome-Wolfson Institute for Experimental Medicine, School of Medicine, Dentistry and Biomedical Sciences, Queen’s University Belfast, 97 Lisburn Road, Belfast, UK.

^4^ Centre for Public Health, School of Medicine, Dentistry and Biomedical Sciences, Queen’s University Belfast, Institute of Clinical Sciences Block B, Belfast UK.

**Corresponding Author:**

Dr Ikhlas A. El Karim

The Wellcome-Wolfson Institute for Experimental Medicine, School of Medicine, Dentistry and Biomedical Sciences, Queen’s University Belfast, 97 Lisburn Road, Belfast, BT9 7BL, United Kingdom

Email: [i.elkarim@qub.ac.uk](mailto:i.elkarim@qub.ac.uk)

Tel: +442890976026

**Supp Table 1: Search Strategy**

| **#** | **Searches** | **Results** |
| --- | --- | --- |
| 1 | copd.mp. [mp=ti, ab, tx, ct, sh, ot, hw, nm, fx, kf, ox, px, rx, an, ui, sy] | 105350 |
| 2 | chronic obstructive pulmonary disease.mp. [mp=ti, ab, tx, ct, sh, ot, hw, nm, fx, kf, ox, px, rx, an, ui, sy] | 136063 |
| 3 | 1 or 2 | 171673 |
| 4 | exacerbation.mp. [mp=ti, ab, tx, ct, sh, ot, hw, nm, fx, kf, ox, px, rx, an, ui, sy] | 131533 |
| 5 | oral bacteria.mp. [mp=ti, ab, tx, ct, sh, ot, hw, nm, fx, kf, ox, px, rx, an, ui, sy] | 6438 |
| 6 | oral health.mp. [mp=ti, ab, tx, ct, sh, ot, hw, nm, fx, kf, ox, px, rx, an, ui, sy] | 55224 |
| 7 | oral hygiene.mp. [mp=ti, ab, tx, ct, sh, ot, hw, nm, fx, kf, ox, px, rx, an, ui, sy] | 44012 |
| 8 | periodontal disease.mp. [mp=ti, ab, tx, ct, sh, ot, hw, nm, fx, kf, ox, px, rx, an, ui, sy] | 41992 |
| 9 | periodontitis.mp. [mp=ti, ab, tx, ct, sh, ot, hw, nm, fx, kf, ox, px, rx, an, ui, sy] | 62141 |
| 10 | gingivitis.mp. [mp=ti, ab, tx, ct, sh, ot, hw, nm, fx, kf, ox, px, rx, an, ui, sy] | 24508 |
| 11 | gingival bleeding.mp. [mp=ti, ab, tx, ct, sh, ot, hw, nm, fx, kf, ox, px, rx, an, ui, sy] | 5887 |
| 12 | porphyromonas gingivalis.mp. [mp=ti, ab, tx, ct, sh, ot, hw, nm, fx, kf, ox, px, rx, an, ui, sy] | 16281 |
| 13 | 5 or 6 or 7 | 90721 |
| 14 | 8 or 9 or 10 or 11 or 12 | 103310 |
| 15 | 3 and 4 | 23887 |
| 16 | 14 and 15 | 170 |
| 17 | 13 and 15 | 155 |
| 18 | hospitalisations.mp. [mp=ti, ab, tx, ct, sh, ot, hw, nm, fx, kf, ox, px, rx, an, ui, sy] | 14397 |
| 19 | hospitalizations.mp. [mp=ti, ab, tx, ct, sh, ot, hw, nm, fx, kf, ox, px, rx, an, ui, sy] | 134158 |
| 20 | 18 or 19 | 148111 |
| 21 | quality of life.mp. [mp=ti, ab, tx, ct, sh, ot, hw, nm, fx, kf, ox, px, rx, an, ui, sy] | 816041 |
| 22 | oral health related quality of life.mp. [mp=ti, ab, tx, ct, sh, ot, hw, nm, fx, kf, ox, px, rx, an, ui, sy] | 4427 |
| 23 | OHRQoL.mp. [mp=ti, ab, tx, ct, sh, ot, hw, nm, fx, kf, ox, px, rx, an, ui, sy] | 2375 |
| 24 | 21 or 22 or 23 | 816057 |
| 25 | cost.mp. [mp=ti, ab, tx, ct, sh, ot, hw, nm, fx, kf, ox, px, rx, an, ui, sy] | 1384775 |
| 26 | expense.mp. [mp=ti, ab, tx, ct, sh, ot, hw, nm, fx, kf, ox, px, rx, an, ui, sy] | 147258 |
| 27 | 25 or 26 | 1481063 |
| 28 | 5 or 6 or 7 or 8 or 9 or 10 or 11 or 12 | 167812 |
| 29 | 3 and 4 and 20 and 28 | 77 |
| 30 | 3 and 4 and 24 and 28 | 118 |
| 31 | 3 and 4 and 27 and 28 | 112 |

**Supp Table 2: excluded studies and reasons for exclusion**

| Reason for exclusion | Number of Articles | Study |
| --- | --- | --- |
| Review Articles | 19 | Mojon 2002 [1]  Dorfer *et al* 2017 [2]  Muthu et al 2016 [3]  Scannapieco *et al* 2003 [4]  Scannapieco *et al* 1999 [5]  Moghadam *et al* 2017 [6]  Sabharwal et al 2018 [7]  Usher *et al* 2013 [8]  Prasanna *et al* 2011 [9]  Scannapieco *et al* 2016 [10]  Linden *et al* 2013 [11]  Azarpazhooh *et al* 2006 [12]  Hobbins *et al* 2017 [13]  Coulthwaite *et al* 2007 [14]  Whisenhunt *et al* 2017 [15]  Bozejac *et al* 2017 [16]  Devlin *et al* 2014 [17]  Tan *et al* 2016 [18]  Shi et al 2018 [19] |
| Outcome Relevance:   - Frequency of exacerbations not assessed - Oral health parameters not assessed. | 15 | Raj *et al* 2014 [20]  Henke *et al* 2016 [21]  Bhavsar *et al* 2015 [22]  Wang *et al* 2009 [23]  Brooke *et al* 2012 [24]  Chung *et al* 2016 [25]  Sharma *et al* 2011 [26]  Gaeckle *et al* 2018 [27]  Tan et al 2019 [28]  Pragman et al 2019 [29]  Shen *et al* 2016 [30]  Prasad *et al* 2020 [31]  Saltnes et al 2015 [32]  Bergstrom et al 2013 [33]  Przybyłowska *et al* 2015 [34] |
| Full Text Unavailable | 3 | Pinto *et al* 2016 [35]  Santos *et al* 2017[36]  Zhou *et al* 2019 [37] |
|  |  |  |

**Supp Table 3 A-** Cross sectional studies

| Adapted quality assessment criteria | *Acceptable (*)* | Liu et *al.* (2012) | AbdelHalim *et al.* (2019) | Zhou *et al.* (2011) |
| --- | --- | --- | --- | --- |
| *Selection:*  Representativeness of sample:  Sample size  Non respondent  Ascertainment of exposure | *Description of sampling strategy*  *Sample justified and satisfactory*  *Comparability between respondents and non-respondents characteristics is*  *established, and the response rate is satisfactory*  *Periodontal parameters measured satisfactorily* | -    -  *  * | -  -  *  * | -  -  *  * |
| *Comparability:*  The subjects in different outcome groups are comparable, based on the study design or analysis. Confounding factors are controlled. | *Controls for the most important factor*  *Control for additional factors* | *  * | -  * | *  * |
| *Outcome:*  Assessment of the outcome | Outcomes appropriately assessed | * | * | * |
| Statistical testing | Appropriate statistical analysis | * | * | * |
| Overall quality Score |  | **6** | **5** | **6** |

**Supp Table 3 B** –Case control studies

| Quality assessment criteria | Acceptable (*) | Baldomero *et al.* (2019) |
| --- | --- | --- |
| *Selection:*  Case definition adequate?  Representativeness of the cases  Selection of the controls  Definition of controls | *All included subjects diagnosed with COPD standard criteria*  *Random sample recruited in a defined setting*  *Controls derived from same population as the cases*  *Controls no history of COPD or exacerbations within defined period* | *  *  *  * |
| *Comparability:*  Comparability of cases and controls on the basis of design and analysis | *Not controlled for the most important factor (smoking)*  *Control for additional factors* | -  * |
| *Exposure:*  Ascertainment of exposure?  Same methods of ascertainment for cases and controls | *Oral health assessment carried out for all cases (clinical/self-reported)*  *Same assessment methods used* | *  * |
| Non Response rate | *Described for participants* | * |
| Overall quality Score |  | **8** |

**Supp Table 3C -** Cohort studies

| Quality assessment criteria | Acceptable (*) | Barros et al. (2013) |
| --- | --- | --- |
| *Selection:*  Representativeness of exposed cohort?  Selection of the non-exposed cohort?  Ascertainment of exposure  Demonstration that outcome of interest is not present at the start of the study | *Sample representative of population*  *Selection of non-exposed cohort*  *Periodontal status and oral health is defined*  *Outcome of interest is not present at the start of the study* | *  *  *  * |
| *Comparability:*  Comparability of cases and controls on the basis of design and analysis | *Control for smoking, age, gender and additional factors* | ** |
| *Outcome*  Assessment of outcome  Was follow up long enough for outcome to occur?  Adequacy of follow up of cohort | *Outcome obtained from records*  *Subjects were followed up for 5 years*  *All subjects accounted for* | *  *  * |
| Overall quality Score |  | **9** |

**Supplementary Table 3**. Quality assessment of observational studies using Newcastle Ottawa Scale (NOS): 3A - adapted NOS for cross-sectional studies, 3B- NOS for case-control studies and 3C NOS for cohort studies. The domains covered by the scale included selection, comparability, outcomes, and exposure. Each asterisk represents whether the individual criterion within the subsection was fulfilled. A maximum of 9 can be assigned for each study using the Newcastle Ottawa Scale. A maximum score of 10 can be assigned for the adapted Newcastle Ottawa Scale for cross sectional studies.

**Scale threshold: Good quality:** 3 or 4 stars in selection domain AND 1 or 2 stars in comparability domain AND 2 or 3 stars in outcome/exposure domain. **Fair quality:** 2 stars in selection domain AND 1 or 2 stars in comparability domain AND 2 or 3 stars in outcome/exposure domain. **Poor quality:** 0 or 1 star in selection domain OR 0 stars in comparability domain OR 0 or 1 stars in outcome/exposure domain.

**Supp Figure 1: Quality assessment of intervention studies using Cochrane RoB 2- tool**

| Study | Bias arising from  the randomization  process | Bias due to deviations  from intended  interventions | Bias due to  missing  outcome data | Bias in  measurement of  the outcome | Bias in selection  of the reported  results | Overall  bias |
| --- | --- | --- | --- | --- | --- | --- |
| Zhou *et al.*  (2014) |  |  |  |  |  |  |
| Agado *et al.* (2012) |  |  |  |  |  |  |

Low risk of bias: Green; High risk of bias: Red; Some concern: Yellow

**Supp Figure 2: Risk of bias assessment of non-randomised intervention studies using ROBINS-I tool**

| Study | Bias due to confounding | Bias in the selection of participants | Bias in the classification of interventions | Bias due to deviations  from intended  interventions | Bias due to missing data | Bias in measurement of outcomes | Bias in selection of the  reported result | Overall risk of bias |
| --- | --- | --- | --- | --- | --- | --- | --- | --- |
| Kucukcokun *et al.* (2013) | Moderate | Low | Low | low | Low | Moderate | Moderate | Moderate |

**Supp reference list**

1. Mojon P. Oral health and respiratory infection. Journal (Canadian Dental Association). 2002.

2. Dörfer C, Benz C, Aida J, Campard G. The relationship of oral health with general health and NCDs: a brief review. International Dental Journal. 2017.

3. Muthu J, Muthanandam S, Mahendra J. Mouth the mirror of lungs: where does the connection lie? Frontiers of Medicine. 2016.

4. Scannapieco FA, Bush RB, Paju S. Associations between periodontal disease and risk for nosocomial bacterial pneumonia and chronic obstructive pulmonary disease. A systematic review. In: Annals of periodontology / the American Academy of Periodontology. 2003.

5. Scannapieco FA. Role of Oral Bacteria in Respiratory Infection. J Periodontol. 1999.

6. Moghadam SA, Shirzaiy M, Risbaf S. The Associations between Periodontitis and Respiratory Disease. Journal of Nepal Health Research Council. 2017.

7. Sabharwal A, Gomes-Filho IS, Stellrecht E, Scannapieco FA. Role of periodontal therapy in management of common complex systemic diseases and conditions: An update. Periodontology 2000. 2018.

8. Usher AKH, Stockley RA. The link between chronic periodontitis and COPD: A common role for the neutrophil? BMC Med. 2013.

9. Prasanna SJ. Causal relationship between periodontitis and chronic obstructive pulmonary disease. J Indian Soc Periodontol. 2011.

10. Scannapieco FA, Cantos A. Oral inflammation and infection, and chronic medical diseases: implications for the elderly. Periodontology 2000. 2016.

11. Linden GJ, Lyons A, Scannapieco FA. Periodontal systemic associations: Review of the evidence. Journal of Clinical Periodontology. 2013.

12. Azarpazhooh A, Leake JL. Systematic Review of the Association Between Respiratory Diseases and Oral Health. J Periodontol. 2006.

13. Hobbins S, Chapple IL, Sapey E, Stockley RA. Is periodontitis a comorbidity of COPD or can associations be explained by shared risk factors/behaviors? International Journal of COPD. 2017.

14. Coulthwaite L, Verran J. Potential pathogenic aspects of denture plaque. British Journal of Biomedical Science. 2007.

15. WHISENHUNT J. Patients who smoke: Statistics still support strong link between tobacco and periodontal disease. RDH. 2017.

16. Bozejac BV, Stojšin I, Đurić M, Zvezdin B, Brkanić T, Budišin E, et al. Impact of inhalation therapy on the incidence of carious lesions in patients with asthma and COPD. J Appl Oral Sci. 2017.

17. Devlin J. Patients with chronic obstructive pulmonary disease: Management considerations for the dental team. Br Dent J. 2014.

18. Tan L, Wang H, Pan C, Zhao J. Periodontal health and chronic obstructive pulmonary disease stratified by smoking: A meta-analysis. Int J Clin Exp Med. 2016.

19. Shi Q, Zhang B, Xing H, Yang S, Xu J, Liu H. Patients with chronic obstructive pulmonary disease suffer from worse periodontal health-evidence from a meta-analysis. Front Physiol. 2018.

20. Raj R, Manu MK, Prakash PY, Singhal DK, Acharya S. The effect of 6 months or longer duration of chronic obstructive respiratory disease medication on the oral health parameters of adults. Spec Care Dent. 2018.

21. Henke C, Budweiser S, Jörres RA. Lung function and associations with multiple dimensions of dental health: A prospective observational cross-sectional study. BMC Res Notes. 2016.

22. Bhavsar NV, Dave BD, Brahmbhatt NA, Parekh R. Periodontal status and oral health behavior in hospitalized patients with chronic obstructive pulmonary disease. J Nat Sci Biol Med. 2015.

23. Wang Z, Zhou X, Zhang J, Zhang L, Song Y, Hu FB, et al. Periodontal health, oral health behaviours, and chronic obstructive pulmonary disease. J Clin Periodontol. 2009.

24. Agado BE, Crawford B, DeLaRosa J, Bowen DM, Peterson T, Neill K, et al. Effects of periodontal instrumentation on quality of life and illness in patients with chronic obstructive pulmonary disease: a pilot study. J Dent Hyg. 2012.

25. Chung JH, Hwang H-J, Kim S-H, Kim TH. Associations Between Periodontitis and Chronic Obstructive Pulmonary Disease: The 2010 to 2012 Korean National Health and Nutrition Examination Survey. J Periodontol. 2016.

26. Kotowycz MA, Syal RP, Afzal R, Natarajan MK, van ’t Hof AW, Liem AL, et al. Reperfusion strategy in Europe: Temporal trends in performance measures for reperfusion therapy in ST-elevation myocardial infarction. Eur Heart J. 2010.

27. Gaeckle NT, Heyman B, Criner AJ, Criner GJ. Markers of dental health correlate with daily respiratory symptoms in COPD. Chronic Obstr Pulm Dis. 2018.

28. Tan L, Tang X, Pan C, Wang H, Pan Y. Relationship among clinical periodontal, microbiologic parameters and lung function in participants with chronic obstructive pulmonary disease. J Periodontol. 2019.

29. Pragman AA, Knutson KA, Gould TJ, Isaacson RE, Reilly CS, Wendt CH. Chronic obstructive pulmonary disease upper airway microbiota alpha diversity is associated with exacerbation phenotype: A case-control observational study. Respir Res. 2019.

30. Shen TC, Chang PY, Lin CL, Chen CH, Tu CY, Hsia TC, et al. Periodontal Treatment Reduces Risk of Adverse Respiratory Events in Patients with Chronic Obstructive Pulmonary Disease. Med (United States). 2016.

31. Prasad B, Nyenhuis SM, Imayama I, Siddiqi A, Teodorescu M. Asthma and Obstructive Sleep Apnea Overlap: What Has the Evidence Taught Us? American journal of respiratory and critical care medicine. 2020.

32. Saltnes SS, Storhaug K, Borge CR, Enmarker I, Willumsen T. Oral health-related quality-of-life and mental health in individuals with chronic obstructive pulmonary disease (COPD). Acta Odontol Scand. 2015.

33. Bergström J, Cederlund K, Dahlén B, Lantz AS, Skedinger M, Palmberg L, et al. Dental Health in Smokers with and without COPD. PLoS One. 2013.

34. Przybyłowska D, Rubinsztajn R, Chazan R, Swoboda-Kopeć E, Kostrzewa-Janicka J, Mierzwińska-Nastalska E. The prevalence of oral inflammation among denture wearing patients with chronic obstructive pulmonary disease. Adv Exp Med Biol. 2015.

35. Pinto EH, Longo PL, De Camargo CCB, Dal Corso S, De Cordoba Lanza F, Stelmach R, et al. Assessment of the quantity of microorganisms associated with bronchiectasis in saliva, sputum and nasal lavage after periodontal treatment: A study protocol of a randomised controlled trial. BMJ Open. 2016.

36. Santos SR, Pinto EH, Longo PL, Dal Corso S, Lanza FC, Stelmach R, et al. Effects of periodontal treatment on exacerbation frequency and lung function in patients with chronic periodontitis: Study protocol of a 1-year randomized controlled trial. BMC Pulm Med. 2017.

37. Zhou X, Liu ZQ, Li W, Wang ZM. [Influence of non-surgical periodontal treatment on the quality of life in chronic obstructive pulmonary disease patients with chronic periodontitis]. Chinese J Stomatol. 2019.

**The PRISMA Check list**

| **Section/topic** | **#** | **Checklist item** | **Reported on page #** |
| --- | --- | --- | --- |
| **TITLE** | | |  |
| Title | 1 | Identify the report as a systematic review, meta-analysis, or both. | 1 |
| **ABSTRACT** | | |  |
| Structured summary | 2 | Provide a structured summary including, as applicable: background; objectives; data sources; study eligibility criteria, participants, and interventions; study appraisal and synthesis methods; results; limitations; conclusions and implications of key findings; systematic review registration number. | 2-3 |
| **INTRODUCTION** | | |  |
| Rationale | 3 | Describe the rationale for the review in the context of what is already known. | 4,5 |
| Objectives | 4 | Provide an explicit statement of questions being addressed with reference to participants, interventions, comparisons, outcomes, and study design (PICOS). | 6 |
| **METHODS** | | |  |
| Protocol and registration | 5 | Indicate if a review protocol exists, if and where it can be accessed (e.g., Web address), and, if available, provide registration information including registration number. | 7 |
| Eligibility criteria | 6 | Specify study characteristics (e.g., PICOS, length of follow-up) and report characteristics (e.g., years considered, language, publication status) used as criteria for eligibility, giving rationale. | 7 |
| Information sources | 7 | Describe all information sources (e.g., databases with dates of coverage, contact with study authors to identify additional studies) in the search and date last searched. | 7 |
| Search | 8 | Present full electronic search strategy for at least one database, including any limits used, such that it could be repeated. | 7 |
| Study selection | 9 | State the process for selecting studies (i.e., screening, eligibility, included in systematic review, and, if applicable, included in the meta-analysis). | 8 |
| Data collection process | 10 | Describe method of data extraction from reports (e.g., piloted forms, independently, in duplicate) and any processes for obtaining and confirming data from investigators. | 9 |
| Data items | 11 | List and define all variables for which data were sought (e.g., PICOS, funding sources) and any assumptions and simplifications made. | 9 |
| Risk of bias in individual studies | 12 | Describe methods used for assessing risk of bias of individual studies (including specification of whether this was done at the study or outcome level), and how this information is to be used in any data synthesis. | 8 |
| Summary measures | 13 | State the principal summary measures (e.g., risk ratio, difference in means). | NA |
| Synthesis of results | 14 | Describe the methods of handling data and combining results of studies, if done, including measures of consistency (e.g., I^2^) for each meta-analysis. | 9 |

Page 1 of 2

| **Section/topic** | **#** | **Checklist item** | **Reported on page #** |
| --- | --- | --- | --- |
| Risk of bias across studies | 15 | Specify any assessment of risk of bias that may affect the cumulative evidence (e.g., publication bias, selective reporting within studies). | 8 |
| Additional analyses | 16 | Describe methods of additional analyses (e.g., sensitivity or subgroup analyses, meta-regression), if done, indicating which were pre-specified. | N/A |
| **RESULTS** | | |  |
| Study selection | 17 | Give numbers of studies screened, assessed for eligibility, and included in the review, with reasons for exclusions at each stage, ideally with a flow diagram. | 9-10 |
| Study characteristics | 18 | For each study, present characteristics for which data were extracted (e.g., study size, PICOS, follow-up period) and provide the citations. | 9  Table 1 |
| Risk of bias within studies | 19 | Present data on risk of bias of each study and, if available, any outcome level assessment (see item 12). | 10 |
| Results of individual studies | 20 | For all outcomes considered (benefits or harms), present, for each study: (a) simple summary data for each intervention group (b) effect estimates and confidence intervals, ideally with a forest plot. | 10-13 |
| Synthesis of results | 21 | Present results of each meta-analysis done, including confidence intervals and measures of consistency. | NA |
| Risk of bias across studies | 22 | Present results of any assessment of risk of bias across studies (see Item 15). | 10 |
| Additional analysis | 23 | Give results of additional analyses, if done (e.g., sensitivity or subgroup analyses, meta-regression [see Item 16]). | N/A |
| **DISCUSSION** | | |  |
| Summary of evidence | 24 | Summarize the main findings including the strength of evidence for each main outcome; consider their relevance to key groups (e.g., healthcare providers, users, and policy makers). | 14-15 |
| Limitations | 25 | Discuss limitations at study and outcome level (e.g., risk of bias), and at review-level (e.g., incomplete retrieval of identified research, reporting bias). | 16 |
| Conclusions | 26 | Provide a general interpretation of the results in the context of other evidence, and implications for future research. | 17 |
| **FUNDING** | | |  |
| Funding | 27 | Describe sources of funding for the systematic review and other support (e.g., supply of data); role of funders for the systematic review. | 18 |

*From:*  Moher D, Liberati A, Tetzlaff J, Altman DG, The PRISMA Group (2009). Preferred Reporting Items for Systematic Reviews and Meta-Analyses: The PRISMA Statement. PLoS Med 6(7): e1000097. doi:10.1371/journal.pmed1000097

For more information, visit: **www.prisma-statement.org**.
